# Supplementary material for: Heat adaptation of phage T7 under an extended genetic code
Source: Virus Evol. 2021 Dec 1;7(2):veab100. doi: 10.1093/ve/veab100 (PMC8923235; doi:10.1093/ve/veab100)
Supplement: veab100_Supp [file veab100_supp.zip › Sequencing_Analysis.zip!/Sequencing_Analysis/S2/evidence/MC_SIDE_2_38.html]

BRESEQ :: Evidence


| Predicted mutation | | | | | | |
| --- | --- | --- | --- | --- | --- | --- |
| evidence | seq id | position | mutation | annotation | gene | description |
| MC JC | NC\_001604.1 | 1,253 | Δ1,489 bp | intergenic (–/–) | – / – | –/– |

| Missing coverage evidence... | | | | | | | | | | |
| --- | --- | --- | --- | --- | --- | --- | --- | --- | --- | --- |
|  |  |  | seq id | start | end | size | ←reads | reads→ | gene | description |
| \* | \* | ÷ | NC\_001604.1 | 1253 | 2741 | 1489 | 5746 [2] | [2] 5753 | *–/–* | –/– |

| New junction evidence | | | | | | | | | | | |
| --- | --- | --- | --- | --- | --- | --- | --- | --- | --- | --- | --- |
|  | | seq id | position | reads (cov) | reads (cov) | score | skew | freq | annotation | gene | product |
| \* | ? | NC\_001604.1 | = 1252 | 2 (0.000) | 5642 (1.100) | 443/580 | 0.0 | 100.0% | intergenic (–/–) | *–/–* | –/– |
|  | ? | NC\_001604.1 | 2742 = | 0 (0.000) |  |  |  |  | intergenic (–/–) | *–/–* | –/– |

Only 100 of 5753 total aligned reads displayed.  

`CAATGATGCCCATTATAAATACGCTGAGATTGCAAGCGACATCATTGATTGCAATTCGGATGAGCATGATGAGTTAACTGGATGGGATGGTGAGTTTGTTGAAACTTGTAAACTAATCCGCAAGTTCTTTGAGGGCATCGCCTCATTCGACATGCATAGCGGGAACATCATGTTCTCAAATGGAGACGTACCATACATCACCGACCCGGTATCATTCTCGCAGAAGAAAGACGGTGGCGCATTCAGCATCGACCCTGAGGAACTCATCAAGGAAGTCGAGGAAGTCGCACGACAGAAAGAAATTGACCGCGCTAAGGCCCGTAAAGAACGTCACGAGGGGCGCTTAGAGGCACGCAGATTCAAACGTCGCAACCGCAAGGCACGTAAAGCACACAAAGCTAAGCGCGAAAGAATGCTTGCTGCGTGGCGATGGGCTGAACGTCAAGAACGGCGTAACCATGAGGTAGCTGTAGATGTACTAGGAAGAACCAATAACGCTATGCTCTGGGTCAACATGTTCTCTGGGGACTTTAAGGCGCTTGAGGAACGAATCGCGCTGCACTGGCGTAATGCTGACCGGATGGCTAT  >  NC_001604.1/2455‑3042`  

                                                                                                                                                                                                                                                                                               `|`                                                                                                                                                                                                                                                                                                              
`cgcttacgaaatgctgaaagaaaacatccgttatgatgacatccgtgacactgatgacctgcacgatgctattcacatggctgccgataatgcagttccgcactactacgctgacatctttagcgtaatggcaagtgagggcattgaccttgagttcgaagactctggtctgatgcctgacaccaaggacgtaatccgcatcctgcaagcgcgtatctatgagcaattaacgattgacctctgggaagacgcagaagacttgctcaatgaatacttggaggaagtcgCACGACagaaagaa                                                                                                                                                                                                                                                                                                 >  1:213215‑M2/288‑301 (MQ=255)`  
  `cttacgaaatgctgaaagaaaacatccgttatgatgacatccgtgacactgatgacctgcacgatgctattcacatggctgccgataatgcagttccgcactactacgctgacatctttagcgtaatggcaagtgagggcattgaccttgagttcgaagactctggtctgatgcctgacaccaaggacgtaatccgcatcctgcaagcgcgtatctatgagcaattaacgattgacctctgggaagacgcagaagacttgctcaatgaatacttggaggaagtcgCACGACAGAAAGAAAt                                                                                                                                                                                                                                                                                               <  1:257640‑M2/16‑1 (MQ=255)`  
      `cgaaatgctgaaagaaaacatccgttatgatgacatccgtgacactgatgacctgcacgatgctattcacatggctgccgataatgcagttccgcactactacgctgacatctttagcgtaatggcaagtgagggcattgaccttgagttcgaagactctggtctgatgcctgacaccaaggacgtaatccgcatcctgcaagcgcgtatctatgagcaattaacgattgacctctgggaagacgcagaagacttgctcaatgaatacttggaggaagtcgCACGACAGAAAGAAATTGAc                                                                                                                                                                                                                                                                                           <  1:236087‑M2/20‑1 (MQ=255)`  
        `aaatgctgaaagaaaacatccgttatgatgacatccgtgacactgatgacctgcacgatgctattcacatggctgccgataatgcagttccgcactactacgctgacatctttagcgtaatggcaagtgagggcattgaccttgagttcgaagactctggtctgatgcctgacaccaaggacgtaatccgcatcctgcaagcgcgtatctatgagcaattaacgattgacctctgggaagacgcagaagacttgctcaatgaatacttggaggaagtcgCACGACAGAAAGAAATTGACcg                                                                                                                                                                                                                                                                                         <  1:472239‑M2/22‑1 (MQ=255)`  
                `caggaaaacatccgttatgatgacatccgtgacactgatgacctgcacgatgctattcacatggctgccgataatgcagttccgcactactacgctgacatctttagcgtaatggcaagtgagggcattgaccttgagttcgaagactctggtctgatgcctgacaccaaggacgtaatccgcatcctgcaagcgcgtatctatgagcaattaacgattgacctctgggaagacgcagaagacttgctcaatgaatacttggaggaagtcgCACGACAGAAAGAAATTGACCGCGCTAAgg                                                                                                                                                                                                                                                                                 <  1:559248‑M2/30‑1 (MQ=255)`  
                 `gagacagcatccgttatgatgacatccgtgacactgatgacctgcacgatgctattcacatggctgccgataatgcagttccgcactactacgctgacatctttagcgtaatggcaagtgagggcattgaccttgagttcgaagactctggtctgatgcctgacaccaaggacgtaatccgcatcctgcaagcgcgtatctatgagcaattaacgattgacctctgggaagacgcagaagacttgctcaatgaatacttggaggaagtcgCACGACAGAAAGAAATTGACCGCGCTAAGGc                                                                                                                                                                                                                                                                                <  1:163366‑M2/31‑1 (MQ=255)`  
                   `gaaaacatccgttatgatgacatccgtgacactgatgacctgcacgatgctattcacatggctgccgataatgcagttccgcactactacgctgacatctttagcgtaatggcaagtgagggcattgaccttgagttcgaggactctggtctgatgcctgacaccaaggacgtaatccgcatcctgcaagcgcgtatctatgagcaattaacgattgacctctgggaagacgcagaagacttgctcaatgaatacttggaggaagtcgCACGACAGAAAGAAATTGACCGCGCTAAGGccc                                                                                                                                                                                                                                                                              >  1:286824‑M2/269‑301 (MQ=255)`  
                       `acatccgttatgatgacatccgtgacactgatgacctgcacgatgctattcacatggctgccgataatgcagttccgcactactacgctgacaactttagcgtaatggcaagtgagggcattgaccttgagttcgaagactctggtctgatgcctgacaccaaggacgtaatccgcatcctgcaagcgcgtatctatgagcaattaacgattgacctctgggaagacgcagaagacttgctcaatgaatacttggaggaagtcgCACGACAGAAAGAAATTGACCGCGCTAAGGCCCGTaa                                                                                                                                                                                                                                                                          <  1:79153‑M2/37‑1 (MQ=255)`  
                                 `tgatgacatccgtgacactgatgacctgcacgatgctattcacatggctgccgataatgcagttccgcactactacgctgacatctttagcgtaatggcaagtgagggcattgaccttgagttcgaagactctggtctgatgcctgacaccaaggacgtaatccgcatcctgcaagcgcgtatctatgagcaattaacgattgacctctgggaagacgcagaagacttgctcaatgaatacttggaggaagtcgCACGACAGAAAGAAATTGACCGCGCTAAGGCCCGTAAAGAACGTCAc                                                                                                                                                                                                                                                                <  1:112911‑M2/47‑1 (MQ=255)`  
                                    `tgacatccgtgacactgatgacctgcacgatgctattcacatggctgccgataatgcagttccgcactactacgctgacatctttagcgtaatggcaagtgagggcattgaccttgagttcgaagactctggtctgatgcctgacaccaaggacgtaatccgcatcctgcaagcgcgtatctatgagcaattaacgattgacctctgggaagacgcagaagacttgctcaatgaatacttggaggaagtcgCACGCCAGAAAGAAATTGACCGCGCTAAAGCCCGTAAAGAACGTCACGAg                                                                                                                                                                                                                                                             >  1:463722‑M2/252‑301 (MQ=255)`  
                                    `tgacatccgtgacactgatgacctccacgatgctattcacatggctgccgataatgcagttccgcactactacgctgacatctttagcgtaatggcaagtgagggcattgaccttgagttcgaagactctggtctgatgcctgacaccaaggacgtaatccgcatcctgcaagcgcgtatctatgagcaattaacgattgacctctgggaagacgcagaagacttgctcaatgaatacttggaggaagtcgCACGACAGAAAGAAATTGACCGCGCTAAGGCCCGTAAAGAACGTCACGAg                                                                                                                                                                                                                                                             <  1:324714‑M2/50‑1 (MQ=255)`  
                                     `aacatccgtgacactgatgacctgcacgatgctattcacatggctgccgataatgcagttccgcactactacgctgacatctttagcgtaatggcaagtgagggcattgaccttgagttcgaagactctggtctgatgcctgacaccaaggacgtaatccgcatcctgcaagcgcgtatctatgagcaattaacgatttacctctgggaagacgcagaagacttgctcaatgaatacttggaggaagtcgCACGACAGAAAGAAATTGACCGCGCTAAGGCCCGTAAAGAACGTCCCGAgg                                                                                                                                                                                                                                                            <  1:530106‑M2/51‑1 (MQ=255)`  
                                                 `actgatgacgtgcacgaggctattcacatgggggccgataatacagttccgcactactacgctcacatctttagcgtaatggcaagtgagggcattgaccgtgagttcgaagactctggtctgatgcctgacaccaaggacgtaatccgcatcctgcaagcgcgtatctatgagcaattaacgattgacctctgggaagacgcagaagacttgctcaatgaatagttggaggaagtcgCACGACAGAAAGAAATTGACCGCGCTAAGGCCCGTAAAGAACGTCACGAGGGGCGCTTAGAgg                                                                                                                                                                                                                                                <  1:294839‑M2/63‑1 (MQ=255)`  
                                                                 `atgctattcacatggctgccgataatgcagttccgcactactacgctgacatctttagcgtaatggcaagtgagggcattgaccttgagttcgaagactctggtctgatgcctgacaccaaggacgtaatccgcatcctgcaagcgcgtatctatgagcaattaacgattgacctctgggaagacgcagaagacttgctcaatgaatacttggaggaagtcgCACGACAGAAAGAAATTGACCGCGCTAAGGCCCGTAAAGAACGTCACGAGGGGCGCTTAGAGGCACGCAGATTCAAACg                                                                                                                                                                                                                                >  1:431051‑M2/223‑301 (MQ=255)`  
                                                                   `gctattcacatggctgccgataatgcagttccgcactactacgctgacatctttagcgtaatggcaagtgagggcattgaccttgagttcgaagactctggtctgatgcctgacaccaaggacgtaatccgcatcctgcaagcgcgtatctatgagcaattaacgattgacctctgggaagacgcagaagacttgctcaatgaatacttggaggaagtcgCACGACAGAAAGAAATTGACCGCGCTAAGGCCCGTAAAGAACGTCACGAGGGGCGCTTAGAGGCACGCAGATTCAAACGTc                                                                                                                                                                                                                              >  1:537796‑M2/221‑301 (MQ=255)`  
                                                                      `attcacatggctgccgataatgcagttccgcactactacgctgacatctttagcgtaatggcaagtgagggcattgaccttgagttcgaagactctggtctgatgcctgacaccaaggacgtaatccgcatcctgcaagtgcgtatctatgagcaattaacgattgacctctgggaagacgcagaagacttgctcaatgaatacttggaggaagtcgCACGACAGAAAGAAATTGACCGCGCTAAGGCCCGTAAAGAACGTCACGAGGGGCGCTTAGAGGCACGCAGATTCAAACGTCGCa                                                                                                                                                                                                                           >  1:757503‑M2/218‑301 (MQ=255)`  
                                                                        `tcacatggctgccgataatgcagttccgcactactacgctgacatctttagcgtaatggcaagtgagggcattgaccttgagttcgaagactctggtctgatgcctgacaccaaggacgtaatccgcatcctgcaagcgcgtatctatgagcaattaacgattgacctctgggaagacgcagaagacttgctcaatgaatacttggaggaagtcgCACGACAGAAAGAAATTGACCGCGCTAAGGCCCGTAAAGAACGTCACGAGGGGCGCTTAGAGGTACGCAGATTCAAACGTCGCAAc                                                                                                                                                                                                                         >  1:83084‑M2/216‑301 (MQ=255)`  
                                                                               `gctgccgataatgcagttccgcactactacgctgacatctttagcgtaatggcaagtgagggcattgaccttgagttcgaagactctggtctgatgcctgacaccaaggacgtaatccgcatcctgcaagcgcgtatctatgagcaattaacgattgacctctgggaagacgcagaagacttgctcaatgaatacttggaggaagtcgCACGACAGAAAGAAATTGACCGCGCTAAGGCCCGTAAAGAACGTCACGAGGGGCGCTTAGAGGCACGCAGATTCAAACGTCGCAACCGCAAgg                                                                                                                                                                                                                  >  1:455250‑M2/209‑301 (MQ=255)`  
                                                                                        `tgtataagagacagctactacgctgacatctttagcgtaatggcaagtgagggcattgaccttgagttcgaagactctggtctgatgcctgacaccaaggacgtaatccgcatcctgcaagcgcgtatctatgagcaattaacgattgacctctgggaagacgcagaagacttgctcaatgaatacttggaggaagtcgCACGACAGAAAGAAATTGACCGCGCTAAGGCCCGTAAAGAACGTCACGAGGGGCGCTTAGAGGCACGCAGATTCAAACGTCGCAACCGCAAGGCACGTAAAg                                                                                                                                                                                                         <  1:447161‑M2/102‑1 (MQ=255)`  
                                                                                           `gcagttccgcactactacgctgacatctttagcgtaatggcaagtgagggcattgaccttgagttcgaagactctggtctgatgcctgacaccaaggacgtaatccgcatcctgcaagcgcgtatctatgagcaattaacgattgacctctgggaagacgcagaagacttgctcaatgaatacttggaggaagtcgCACGACAGAAAGAAATTGACCGCGCTAAGGCCCGTAAAGAACGTCACGAGGGGCGCTTAGAGGCACGCAGATTCAAACGTCGCAACCGCAAGGCACGTAAAGcac                                                                                                                                                                                                      <  1:18693‑M2/105‑1 (MQ=255)`  
                                                                                              `gttccgcactactacgctgacatctttagcgtaatggcaagtgagggcattgaccttgagttcgaagactctggtctggtgcctgacaccaaggacgtaatccgcatcctgcaagcgcgtatctatgcgcaattaacgattgacctctgggaagacgcagaagacttgctcaatgaatacttggaggaagtcgCACGACAGCAAGAAATTGACCGCGCTAAGGCCCGTAAAGAACGTCACGAGGGGCGCTTAGAGGCACGCAGATTCCAACGTCCCACACGCAAGGCACGTAAAGaacaca                                                                                                                                                                                                   >  1:157604‑M2/194‑301 (MQ=255)`  
                                                                                                 `ccgcactactacgctgacatctttagcgtaatggcaagtgagggcattgaccttgagttcgaagactctggtctgatgcctgacaccaaggacgtaatccgcatcctgcaagcgcgtatctatgagcaattaacgattgacctctgggaagacgcagaagacttgctcaatgaatacttggaggaagtcgCACGACAGAAAGCAATTGACCGCGCTAAGGCCCGTAAAGCACGTCACGAGGGGCGCTTAGAGGCACGCATATTCAAACGTCGCCACCGCAAGGCACCTAAAGCACACTAAg                                                                                                                                                                                                >  1:713814‑M2/191‑301 (MQ=255)`  
                                                                                                      `ctactacgctgacatctttagcgtaatggcaagtgagggcattgaccttgagttcgaagactctggtctgatgcctgacaccaaggacgtaatccgcatcctgcaagcgcgtatctatgagcaattaacgattgacctctgggaagacgcagaagacttgctcaatgaatacttggaggaagtcgCCCGACAGAAAGAAATTGACCGCGCTAAGGCCCGTAAAGAACGTCACGAGGGGCGCTTAGAGGCACGCCGTTTCAAACGTCGCAACCGCACGGCACGTACAGCACACAAAGCTAAg                                                                                                                                                                                           >  1:250911‑M2/186‑301 (MQ=255)`  
                                                                                                      `ctactacgctgacatctttagcgtaatggcaagtgagggcattgaccttgagttcgaagactctggtctgatgcctgacaccaaggacgtaatccgcatcctgcaagcgcgtatctatgagcaattaacgattgacctctgggaagacgcagaagacttgctcaatgaatacttggaggaagtcgCACGACAGAAAGAAATTGACCGCGCTAAGGCCCGTAAAGAACGTCACGAGGGGCGCTTAGAGGCACGCAGAttcaaacgtcgcaaccgcaaggcctgtctcttatacacatctccc                                                                                                                                                                                           >  1:799936‑M2/186‑279 (MQ=255)`  
                                                                                                      `ctactacgctgacatctttagcgtaatggcaagtgagggcattgaccttgagttcgaagactctggtctgatgcctgacaccaaggacgtaatccgcatcctgcaagcgcgtatctatgagcaattaacgattgacctctgggaagacgcagaagacttgctcaatgaatacttggaggaagtcgCACGACAGAAAGAAATTGACCGCGCTAAGGCCCGTAAAGAACGTCACGAGGGGCGCTTAGAGGCACGCAGATTCAAACGTCGCAACCGCAAGGCACGTCAAGCACACAAAGCTAAg                                                                                                                                                                                           >  1:572813‑M2/186‑301 (MQ=255)`  
                                                                                                        `actacgctgacatctttagcgtaatggcaagtgagggcattgaccttgagttcgaagactctggtctgatgcctgacaccaaggacgtaatccgcatcctgcaagcgcgtatctatgagcaattaacgattgacctctgggaagacgcagaagacttgctcaatgaatacttggaggaagtcgCACGACAGAAAGAAATTGACCGCGCTAAGGCCCGTAAAGAACGTCACGAGGGGCGCTTAGAGGCACGCAGATTCAAACGTCGCAACCGCAAGGCACGTAAAGCACacaaagcccgtcc                                                                                                                                                                                         >  1:350017‑M2/184‑295 (MQ=255)`  
                                                                                                           `acgctgacatctttagcgtaatggcaagtgagggcattgaccttgagttcgaagactctggtctgatgcctgacaccaaggacgtaatccgcgtcctgcaagcgcgtatctatgagcaattaacgattgacctctgggaagacgcagaagacttgctcaatgaatacttggaggaagtcgCACGACAGAAAGAAATTGACCGCGCTAAGGCCCGTAAAGAACGTCAAGAGGGGCGCTTAGAGGCACGCAGATTCAAACGTCGCAACCGCAAGGCACGTAAAGCACACCAAGCTAAgcgcgg                                                                                                                                                                                      >  1:660078‑M2/181‑300 (MQ=255)`  
                                                                                                           `acgctgacatctttagcgtaatggcaagtgagggcattgaccttgagttcgaagactctggtctgatgcctgacaccaaggacgtaatccgcatcctgcaagcgcgtatctatgagcaattaacgattgacctctgggaagacgcagaagacttgctcaatgaatacttggaggaagtcgCACGACAGAAAGAAATTGACCGCGCTAAGGCCCGTAAAGAACGTCACGAGGGGCGCTTAGAGGCACGCAGATTCAAACGTCGCAACCGCAAGGCACGTAAAGCACACAAAGCTAAGCGCGa                                                                                                                                                                                      >  1:615921‑M2/181‑301 (MQ=255)`  
                                                                                                                `gacatctttagcgtaatggcaagtgagggcattgaccttgagttcgaagactctggtctgatgcctgacaccaaggacgtaatccgcatcctgcaagcgcgtatctatgagcaattaacgattgacctctgggaagacgcagaagacttgctcaatgaatacttggaggaagtcgCACGACAGAAAGAAATTGACCGCGCTAAGGCCCGTAAAGAACGTCACGAGGGGCGCTTAGAGGCACGCAGATTCAAACGTCGCAACCGCAAGGCACGTAAAGCACACAAAGCTAAGCGcgaacgaa                                                                                                                                                                                 >  1:106593‑M2/176‑301 (MQ=255)`  
                                                                                                                `gacatctttagcgtaatggcaagtgagggcattgaccttgagttcgaagactctggtctgatgcctgacaccaaggacgtaatccgcatcctgcaagcgcgtatctatgagcaattaacgattgacatctgggaagacgcagaagagttgctcaatgaatacttggaggaagtcgCACGACAGAAAGAAATTGACCGCGCTATGGCCCGTGAAGAACGTCACGAGGGGGGATTAGAGGGACGCAGATTCAAACGTCGCAACAGGAAGGCACGTAAAGCACACAAAGCTAAGGGCGACAGaa                                                                                                                                                                                 >  1:681729‑M2/176‑301 (MQ=255)`  
                                                                                                                   `atctttagcgtaatggcaagtgagggcattgaccttgagttcgaagactctggtctgatgcctgacaccaaggacgtaatccgcatcctgcaagcgcgtatctatgagcaattaacgattgacctctgggaagacgcagaagacttgctcaatgaatacttggaggaagtcgCACGACAGAAAGAAATTGACCGCGCTAAGGCCCGTAAAGAACGTCACGAGGGGCGCTTAGAGGCACGCAGATTCAAACGTCGCAACCGCAAGGCACGTAAAGCACACAAAGCTAAGCGCGAAAGAAtgc                                                                                                                                                                              <  1:378866‑M2/129‑1 (MQ=255)`  
                                                                                                                   `atctttagcgtaatggcaagtgagggaattgaccttgagttcgaagactctggtctgatgcctgacaccaaggacgtaatccgcatcctgcaagcgcgtatctatgagcaattaacgattgacctctgggaagacgcagaagacttgctcaatgaatacttggaggaagtcgCACGACAGAAAGAAATTGACCGCGCTAAGGCCCGTAAAGAACGTCACGAGGGGCGCTTAGAGGCACGCAGATTCAAACGTCGCAACCGCAAGGCACGTAAAGCACACAAAGCTAAGCGCGAAAGAAtgc                                                                                                                                                                              <  1:806359‑M2/129‑1 (MQ=255)`  
                                                                                                                          `gcgtaatggcaagtgagggcattgaccttgagttcgaagactctggtctgatgcctgacaccaaggacgtaatccgcatcctgcaagcgcgtatctatgagcaattaacgattgacctctgggaagacgcagaagacttgctcaatgaatacttggaggaagtcgCACGACAGAAAGAAATTTACCGCGCTAAGGCCCGTAAAGAACGTCACGAGGGGCGCTTATAGGCACGCAGATTCAAACGTCGAAACCGCAAGGCACGGAAAGCACACAAAGATAAGCGCGAAAGAATGCTtgctgc                                                                                                                                                                       >  1:771898‑M2/166‑301 (MQ=255)`  
                                                                                                                            `gtaatggcaagtgagggcattgaccttgagttcgaagactctggtctgatgcctgacaccaaggacgtaatccgcatcctgcaagcgcgtatctatgagcaattaacgattgacctctgggaagacgcagaagacttgctcaatgaatacttggaggaagtcgCACGACAGAAAGAAATTGACCGCGCTAAGGCCCGTAAAGAACGTCACGAGGGGCGCTTAGAGGCACGCAGATTCAAACGTCGCAACCGCAAGGCACGTAAAGCACACAAAGCTAAGCGCGAAAGAATGCTTGCTGcgg                                                                                                                                                                     >  1:358250‑M2/164‑300 (MQ=255)`  
                                                                                                                               `gtggcaagtgagggcattgaccttgagttcgaagactctggtctgatgcctgacaccaaggacgtaatccgcatcctgcaagcgcgtatctatgagcaattaacgattgacctctgggaagacgcagaagacttgctcaatgaatacttggaggaagtcgCACGACAGAAAGAAATTGACCGCGCTAAGGCCCGTAAAGAACGTCACGAGGGGCGCTTAGAGGCACGCAGATTCAAACGTCGCAACCGCAAGGCACGTAAAGCACACAAAGCTAAGCGCGAAAGAATGCTTGCTGCGTGGc                                                                                                                                                                  <  1:56559‑M2/141‑1 (MQ=255)`  
                                                                                                                               `atggcaagtgagggcattgaccttgagttcgaagactctggtctgatgcctgacaccaaggacgtaatccgcatcctgcaagcgcgtatctatgagcaattaacgattgatctctgggaagacgcagaagacttgctcaatgaatacttggaggaagtcgCACGACAGAAAGAAATTGACCGCGCTAAGGCCCGTAAAGAACGTCACGAGGGGCGCTTAGAGGCACGCAGATTCAAACGTCGCAACCGCAAGGCACGTAAAGCACACAAAGCTAAGCGCGAAAGAATGCTTGCTGCGTGGc                                                                                                                                                                  >  1:666036‑M2/161‑301 (MQ=255)`  
                                                                                                                               `atggcaagtgagggcattgaccttgagttcgaagactctggtctgatgcctgacaccaaggacgtaatccgcatcctgcaagcgcgtatctatgagcaattaacgattgacctctgggaagacgcagaagacttgctcaatgaatacttggaggaagtcgCACGACAGAAAGAAATTGACCGCGCTAAGGCCCGTAAAGAACGTCACGAGGGGCGCTTAGAGGCACGCAGATTCAAACGTCGCAACCGAAAGGCACGTAAAGCACACCAAGCCAAGCGCGAAAGAATGCTTGCTGCGtggg                                                                                                                                                                  >  1:765637‑M2/161‑300 (MQ=255)`  
                                                                                                                                 `gtataagagacagattgaccttgagttcgaagactctggtctgatgcctgacaccaaggacgtaatccgcatcctgcaagcgcgtatctatgagcaattaacgattgacctctgggaagacgcagaagacttgctcaatgaatacttggaggaagtcgCACGACAGAAAGAAATTGACCGCGCTAAGGCCCGTAAAGAACGTCACGAGGGGCGCTTAGAGGCACGCAGATTCAAACGTCGCAACCGCAAGGCACGTAAAGCACACAAAGCTAAGCGCGAAAGAATGCTTGCTGCGTGGCGa                                                                                                                                                                <  1:100099‑M2/143‑1 (MQ=255)`  
                                                                                                                                      `gtgagggcattgaccttgagttcgaagactctggtctgatgcctgacaccaaggacgtaatccgcatcctgcaagcgcgtatctatgagcaattaacgattgacctctgggaagacgcagaagacttgctcaatgaatacttggaggaagtcgCACGACAGAAAGAAATTGACCGCGCTAAGGCCCGTAAAGAACGTCACGAGGGGCGCTTAGAGGCACGCAGATTCAAACGTCGCAACCGCAAGGCACGTAAAGCACACAAAGCTAAGCGCGAAAAAATGCTTGCTACGTGGCGATGGGc                                                                                                                                                           >  1:54394‑M2/154‑301 (MQ=255)`  
                                                                                                                                           `tgcattgaccttgagttcgaagactctggtctgatgcctgacaccaaggacgtaatccgcatcctgcaagcgcgtatctattagcaattaacgattgacctctgggaagacgcagaagacttgctcaatgaatacttggaggaagtcgCACGACAGAAAGAAATTGACCGCGCTAAGGCCCGTAAAGAACGTCACGAGGGGCGCTTAGAGGCACGCAGATTCAAACGTCGCAACCGCAAGGCACGTAAAGCACACAAAGCTAAGCGCGAAAGAATGCTTGCTGCGTGGCGATGGGCTGAAc                                                                                                                                                      <  1:698534‑M2/153‑1 (MQ=255)`  
                                                                                                                                           `ggcattgaccttgagttcgaagactctggtctgatgcctgacaccaaggacgtaatccgcatcctgcaagcgcgtatctatgagcaattaacgattgacctctgggaagacgcagaagacttgctcaatgaatacttggaggaagtcgCACGACAGAAAGAAATTGACCGCGCTAAGGCCAGTAAAGAACGTCACGAGGGGCGCTTAGAGGCACGCAGATTCAAACGTCGCAACCGCAAGGCACGTAAAGAACACAAAGCTAAGCGCGAAAGAATGCTTGCTGCGTGGCGATGGGCTGAAc                                                                                                                                                      >  1:386498‑M2/149‑301 (MQ=255)`  
                                                                                                                                            `gcatttacctttagttctaagactctgggatgatgcctgaccccaaggacgtaatccgcatcctgcaagcgcgtatctataagcaattaacgattgacctctgggaagacgcagaagacttgctcaatgaatacttggaggaggtcgCACGACAGAAAGAAATTGACCGCGCTAAGGCCCGTAAAGAACGTCACGAGTGGCGCTTAGAGGCACGCAGATTCAAACGTCGCAACCGCAAGGCACGTAAAGCACACAAAGCTAAGCGCGAAAGAATGCTTGCTGCGTGGCGATGGGCTAAACg                                                                                                                                                     <  1:207612‑M2/154‑1 (MQ=255)`  
                                                                                                                                            `gcattgaccttgagttcgaagactctggtctgatgcctgacaccaaggacgtaatccgcatcctgcaagcgcgtatctatgagcaattaacgattgacctctgggaagacgcagaagacttgctcaatgaatacttggaggaagtcgCACGACAGAAAGAAATTGACCGCGCTAAGGCCCGTAAAGAACGTCACGAGGGGCGCTTAGAGGCACGCAGATTCAAACGTCGCAACCGCAAGGCACGTAAAGCACACAAAGCTAAGCGCGAAAGAATGCTTGCTGCGTGGCGATGGGCTGAACg                                                                                                                                                     >  1:48598‑M2/148‑301 (MQ=255)`  
                                                                                                                                            `gcattgaccttgagttcgaagactctggtctgatgcctgacaccaaggacgtaatccgcatcctgcaagcgcgtatctatgagcaattaacgattgacctctgggaagacgcagaagacttgctcaatgaatacttggaggaagtcgCACGACAGAAAGAAATTGACCGCGCTAAGGCCCGTAAAGAACGTCACGAGGGGCGCTTAGAGGCACGCAGATTCAAACGTCGCAACCGCAAAGCACGTAAAGCACACAAAGCTAAGCGCGAAAGAATGCTTGCTGCGTGGCGATGGGCTGAACg                                                                                                                                                     >  1:128564‑M2/148‑301 (MQ=255)`  
                                                                                                                                             `cattgaccttgagttcgaagactctggtctgatgcctgacaccaaggacgtaatccgcatcctgcaagcgcgtatctatgagcaattaacgattaacctctgggaagacgcagaagacttgctcaatgaatacttggaggaagtcgCACGACAGAAAGAAATTGACCGCGCTAAGGCCCGTAAAGAACGTCACGAGGGGCGCTTAGAGGCACGCAGATTCAAACGTCGCAACCGCAAGGCACGTAAAGCACACAAAGCTAAGCGCGAAAGAATGCTTGCTGCGTGGCGATGGGCTGAACGt                                                                                                                                                    <  1:736781‑M2/155‑1 (MQ=255)`  
                                                                                                                                                 `gaccttgagttcgaagactctggtctgatgcctgacaccaaggacgtaatccgcatcctgcaagcgcgtatctatgagcaattaacgattgacctctgggaagacgcagaagacttgctcaatgaatacttggaggaagtcgCACGACAGAAAGAAATTGACCGCGCTAAGGCCCGTAAAGAACGTCACGAGGGGCGCTTAGAGGCACACAGATTCAAACGTCGCAACCGCAAGGCACGTAAAGCACACAAAGCTAAGCGCGAAAGAATGCTTGCTGCGTGGCGATGGGCTGAACGTCAAg                                                                                                                                                <  1:551572‑M2/159‑1 (MQ=255)`  
                                                                                                                                                       `gagttcgaagactctggtctgatgcctgacaccaaggacgtaatccgcatcctgcaagcgcgtatctatgagcaattaacgattgacctctgggaagacgcagaagacttgctcaatgaatacttggaggaagtcgCACGACAGAAAGAAATTGACCGCGCTAAGGCCCGTAAAGAACGTCACGAGGGGCGCTTAGAGGCACGCAGATTCAAACGTCGCAACCGCAAGGCACGTAAAGCACACAAAGCTAAGCGCGAAAGAATGCTTGCTGCGTGGCGATGGGCTGAACGTCAAgaacctg                                                                                                                                          >  1:91582‑M2/137‑298 (MQ=255)`  
                                                                                                                                                       `gagttcgaagactctggtctgatgcctgacaccaaggacgtaatccgcatcctgcaagcgcgtatctatgagcaattaacgattgacctctgggaagacgcagaagacttgctcaatgaatacttggaggaagtcgCACGACAGAAAGAAATTGACCGCGCTAAGGCCCGTAAAGAACGTCACGAGGGGCGCTTAGAGGCACGCAGATTCAAACGTCGCAACCGCAAGGCACGTAAAGCACACAAAGCTAAGCGCGAAAGAATGCTTGCTGCGTGGCGATGGGCTGAACGTCAAGAACGGc                                                                                                                                          >  1:49982‑M2/137‑301 (MQ=255)`  
                                                                                                                                                         `gttcgaagactctggtctgatgcctgacaccaaggacgtaatccgcatcctgcaagcgcgtatctatgagcaatgaacgattgacctctgggaagacgcagaagacttgctcaattaatacttggaggaagtcgCACGACAGAAAGAAATTGACCGCGCTAAGGCCCGTAAAGAACGTCACGAGGGGCGCTTAGAGGCACGGAGATTCAAACGTCGCAACCGCAAGGCACGTAAAGCACACAAAGCTAAGCGCGAAAGAATGCTTGCTGCGTGGCGATGGGCTGAACGTCAAGCACGGcgg                                                                                                                                        >  1:243074‑M2/135‑300 (MQ=255)`  
                                                                                                                                                             `gaagactctggtctgatgcctgacaccaaggacgtaatccgcatcctgcaagcgcgtatctatgagcaattaacgattgacctctgggaagacgcagaagacttgctcaatgaatacttggaggaagtcgCACGACAGAAAGAAATTGACCGCGCTAAGTCCCGTAAAGAACGTCACGAGGGTCGCTTAGAGGCACGCAGATTCAAACGTCGCAACCGCAAGGCACGTAAAGCACACAAAGCTAAGCGCGAAAGAATGCTTGCTGCGTGGCGATGGGCTGAACGTCAAGAACGGCGTAAcc                                                                                                                                    <  1:366043‑M2/171‑1 (MQ=255)`  
                                                                                                                                                              `agagacagggtctgatgcctgaacccaaggacgtaatccgcatccggcaagcgcgtatctatgagcaattaacgattgacctctgggaagacgcagaagacttgctcaatgaatacttggaggaagtcgCACGACAGAAAGAAATTGACCGCGCTAAGGCCCGTAAAGAACGTCACGAGGGGCGCTTAGAGGCACGCAGATTCAAACGTCGCAACCGCAAGGCACGTAAAGCACACAAAGCTAAGCGCGAAAGAATGCTTGCTGCGTGGCGATGGGCTGAACGTCAAGAACGGCGTAACCa                                                                                                                                   <  1:81426‑M2/172‑1 (MQ=255)`  
                                                                                                                                                                   `aagggtctgatgcctgacaccaaggacgtaatccgcatcctgcaagcgcgtatctatgagcaattaacgattgacctctgggaagacgcagaagacttgctcaatgaatacttggaggaagtcgCACGACAGAAAGAAATTGACCGCGCTAAGGCCCGTAAAGAACGTCACGAGGGGCGCTTAGAGGCACGCAGATTCAAACGTCGCAACCGCAAGGCACGTAAAGCACACAAAGCTAAGCGAGAAAGAATGCTTGCTGCGTGGCGATGGGCTGAACGTCAAGAACGGCGTAACCATGAgg                                                                                                                              <  1:120728‑M2/177‑1 (MQ=255)`  
                                                                                                                                                                      `ggtctgatgcctgacaccaaggacgtaatccgcatcctgcaagcgcgtatctatgagcaattaacgattgacctctgggaagacgcagaagacttgctcaatgaatacttggaggaagtcgCACGACAGAAAGAAATTGACCGCGCTAAGGCCCGTAAAGAACGTCACGAGGGGCGCTTAGAGGCACGCAGATTCAAACGTCGCAACCGCAAGGCACGTAAAGCACACAAAGCTAAGCGCGAAAGAATGCTTGCTGCGTGGCGATGGGCTGAACGTCAAGAACGGCGTAACCATGAGGTAg                                                                                                                           >  1:586999‑M2/122‑301 (MQ=255)`  
                                                                                                                                                                      `ggtctgatgcctgacaccaaggacgtaatccgcatcctgcaagcgcgtatctatgagcaattaacgattgacctctgggaagacgcagaagacttgctcaatgaatacttggaggaagtcgCACGACAGAAAGAAATTGACCGCGCTAAGGCCCGTAAAGAACGTCACGAGGGGCGCTTAGAGGCACGCAGATTCAAACGTCGCAACCGCAAGGCACGTAAAGCACACAAAGCTAAGCGCGAAAGAATGCTTGCTGCGTGGCGATGGGCTGAACGTCAAGAACGGCGTAACCATGAGATAg                                                                                                                           >  1:170968‑M2/122‑301 (MQ=255)`  
                                                                                                                                                                       `gtctgatgcctgacaccaaggacgtaatccgcatcctgcaagcgcgtatctatgagcaattaacgattgacctctgggaagacgcagaagacttgctcaatgaatacttggaggaagtcgCACGACAGAAAGAAATTGACCGCGCTAAGGCCCGTAAAGAACGTCACGAGGGGCGCTTAGAGGCACGCAGATTCAAACGTCGCAACCGCAAGGCACGTAAAGCACACAAAGCTAAGCGCGAAAGAATGCTTGCTGCGTGGCGATGGGCTGAACGTCAAGAACGGCGTAACCATGAAGTAGc                                                                                                                          >  1:779340‑M2/121‑301 (MQ=255)`  
                                                                                                                                                                         `ctgatgcctaacaccaaggacgtactccgcatcctgcaagcgcgtatctatgagcaattaacgattgaactctgggaagacgcagaagacttgctcaatgaatacttggaggaagtcgCACGACAGAAAGAAATTGACCGAGCTAAGGCCCGTAAAGAACGTCACGAGGGGCGCTTAGAGGCACGCATATTCAAACGTCGCAACCGCAAGGCACGTAAAGCACACAAAGCTAAGCGCGAAAGAATGCTTGCTGCGTGGCGATGGGCTGAACGTCAAGAACGGCGTAACCATGAGGTAGCTg                                                                                                                        <  1:51395‑M2/183‑1 (MQ=255)`  
                                                                                                                                                                           `ataagagacagccaaggacgtaatccgcatcctgcaagcgcgtatctatgagcaattaacgattgacctctgggaagacgcagaagacttgctcaatgaatacttggaggaagtcgCACGACAGAAAGAAATTGACCGCGCTAAGGCCCGTAAAGAACGTCACGAGGGGCGCTTAGAGGCACGCAGATTCAAACGTCGCAACCGCAAGGCACGTAAAGCACACAAAGCTAAGCGCGAAAGAATGCTTGCTGCGTGGCGATGGGCTGAACGTCAAGAACGGCGTAACCATGAGGTAGCTGTa                                                                                                                      <  1:439537‑M2/185‑1 (MQ=255)`  
                                                                                                                                                                              `gcctgacaccaaggacgtaatccgcatcctgcaagcgcgtatctatgagcaattaacgattgacctctgggaagacgcagaagacttgctcaatgaatacttggaggaagtcgCACGACAGAAAGAAATTGACCGCGCTAAGGCCCGTAAAGAACGTCACGAGGGGCGCTTAGAGGCACGCAGATTCAAACGTCGCAACCGCAAGGCACGTAAAGCACACAAAGCTCAGCGCGCAAGAATGCTTGCTGCGTGGCGATGGGCTGAACGTCACGCACGGCGTAACCATGAGGTAGCTGTAGAt                                                                                                                   >  1:178076‑M2/114‑301 (MQ=255)`  
                                                                                                                                                                              `gcctgacaccaaggacgtaatccgcatcctgcaagcgcgtatctatgagcaattaacgattgacctctgggaagacgcagaagacttgctcaatgaatacttggaggaagtcgCACGACAGAAAGAAATTGACCGCGCTAAGGCCCGTAAAGAACGTCACGAGGGGCGCTTAGAGGCACGCAGATTCAAACGTCGCAACCGCAAGGCACGTAAAGCACACAAAGCTAAGCGCGAAAGAATGCTTGCTGCGTGGCGATGGGCTGAACGTCAAGAACGGCGTAACCATGAGGTAGCTGTAGAt                                                                                                                   <  1:279990‑M2/188‑1 (MQ=255)`  
                                                                                                                                                                               `cctgacaccaaggacgtaatccgcatcctgcaagcgcgtatctatgagcaattaacgattgacctctgggaagacgcagaagacttgctcaatgaatacttggaggaagtcgCACGACAGAAAGAAATTGACCGCGCTAAGGCCCGTAAAGAACGTCACGAGGGGCGCTTAGAGGCACGCAGATTCAAACGTCGCAACCGCAAGGCACGTAAAGCACACAAAGCTAAGCGCGAAAGAATGCTTGCTGCGTGGCGATGGGCTGAACGTCAAGAACGGCGTAACCATGAGGTAGCTGTAGATg                                                                                                                  <  1:142982‑M2/189‑1 (MQ=255)`  
                                                                                                                                                                               `caggacaccatgtacggaatccgcagccggcaagcgctgatctattagcaagtaacgatggacccctgggaagacgcagaagacgtgctcaatgaaaacttggaggaagtcgCAAGACAGAAAGAAATTGACCGCGCTAAGGCCCGTAAAGAACGTCACGAGGGGCGCTTAGAGTCACGCAGATTCAAACGTCGCAACCGCAAGGCACGTAAAGCACACAAAGCTAAGCGCGAAAGAAGGCTTGCGGCGTGGCGAGGTGCTGAACGGCAAGAACGGCGTAACCATGAGGTAGCTGTAGATg                                                                                                                  <  1:62547‑M2/189‑1 (MQ=255)`  
                                                                                                                                                                                  `gacaccaaggacgtaatccgcatcctgcaagcgcgtatctatgagcaattaacgattgacctctgggaagacgcagaagacttgctcaatgaatacttggaggaagtctCACGACAGAAAGAAATTGACCGCGCTAAGGCCCGTAAAGAACGTCACGAGGGGCGCTTAGAGGCACGCAGATTCAAACGTCGCAACCGCAAGGCACGTAAAGCACACAAAGCTAAGCGCGAAAGAATGCTTGCTGCGTGGCGATGGGCTGAACGTCAAGAACGGCGTAACCATGAGGTAGCTGTAGATGTAc                                                                                                               >  1:689450‑M2/110‑301 (MQ=255)`  
                                                                                                                                                                                  `gacaccaaggacgtaatccgcatcctgcaagcgcgtatctatgagcaattaacgattgacctctgggaagacgcagaagacttgctcaatgaatacttggaggaagtcgCACGACAGAAAGAAATTGACCGCGCTAAGGCCCGTAAAGAACGTCACGAGGGGCGCTTAGAGGCACGCAGATTCAAACGTCGCAACCGCAAGGCACGTAAAGCACACAAAGCTAAGCGCGAAAGAATGCTTGCTGCGTGGCGATGGGCTGAACGTCAAGAACGGCGTAACTATGAGGTAGCTGTAGATGTAc                                                                                                               >  1:423484‑M2/110‑301 (MQ=255)`  
                                                                                                                                                                                   `acaccaaggacgtaatccgcatcctgcaagcgcgtatctatgagcaattaacgattgacctctgggaagacgcagaagacttgctcaatgaatacttggaggaagtcgCACGACAGAAAGAAATTGACCGCGCTAAGGCCCGTAAAGAACGTCACGAGGGGCGCTTAGAGGCACGCAGATTCAAACGTCGCAACCGCAAGGCACGTAAAGCACACAAAGCTACGATCGAAAGAATGCTTGCTGCGTGGCGCTGGGCTGAACGTCAAGAACGGCGGAACCATTAAGTATATGTATATGTACt                                                                                                              >  1:150838‑M2/109‑301 (MQ=255)`  
                                                                                                                                                                                    `caccaaggacgtaatccgcatcctgcaagcgcgtatctatgagcaattaacgattgacctctgggaagacgcagaagacttgctcaatgaatacttggaggaagtcgCACGACAGAAAGAAATTGACCGCGCTAAGGCCCGTAAAGAACGTCACGAGGGGCGCTTAGAGGCACGCAGATTCAAACGTCGCAACCGCAAGGCACGTAAAGCACACAAAGCTAAGCGCGAAAGAATGCTTGCTGCGTGGCGATGGGCTGAACGTCAAGAACGGCTTAACCATGAGGTAGCTGTAGTTGTActg                                                                                                             >  1:609256‑M2/108‑300 (MQ=255)`  
                                                                                                                                                                                    `caccaaggacgtaatccgcatcctgcaagcgcgtatctatgagcaattaacgattgacctctgggaagacgcagaagacttgctcaatgaatacttggaggaagtcgCACGACAGAAAGAAATTGACCGCGCTAAGGCCCGTAAAGAACGTCACGAGGGGCGCTTAGAGGCACGCAGATTCAAACGTCGCAACCGCAAGGCACGTAAAGCACACAAAGCTAAGCGCGAAAGAATGCTTGCTGCGTGGCGATGGGCTGAACGTCAAGAACGGCGTAACCATGAGGTAGCTGTAGATGTACTa                                                                                                             >  1:729685‑M2/108‑301 (MQ=255)`  
                                                                                                                                                                                    `caccaaggacgtaatccgcatcctgcaagcgcgtatctatgagcaattaacgattgacctctgggaagacgcagaagacttgctcaatgaatacttggaggaagtcgCACGACAGAAAGAAATTGACCGCGCTAAGGCCCGTAAAGAACGTCACGAGGGGCGCTTAGAGGCACGCAGATTCAAACGTCGCAACCGCAAGGCACGTAAAGCACACAAAGCTAAGCGCGAAAGAATGCTTGCTGCGTGGAGATGGGCTGAACGTCAAGAACGGCGTAACCATGAGGTAGCtgtagatctgtct                                                                                                             >  1:649922‑M2/108‑295 (MQ=255)`  
                                                                                                                                                                                       `aagagacagaatccgcatcctgcaagcgcgtatctatgagcaattaacgattgacctctgggaagacgcagaagacttgctcaatgaatacttggaggaagtcgCACGACAGAAAGAAATTGACCGCGCTAAGGCCCGTAAAGAACGTCACGAGGGGCGCTTAGAGGCACGCAGATTCAAACGTCGCAACCGCAAGGCACGTAAAGCACACAAAGCTAAGCGCGAAAGAATGCTTGCTGCGTGGCGATGGGCTGAACGTCAAGAACGGCGTAACCATGAGGTAGCTGTAGATGTACTAGga                                                                                                          <  1:722557‑M2/197‑1 (MQ=255)`  
                                                                                                                                                                                          `ggacgtaatccgcatcctgcaagcgcgtatctatgagcaattaacgattgacctctgggaagacgcagaagacttgctcaatgaatacttggaggaagtcgCAAGACAGAAAGAAATTGACCGCGCTAAGGCCCGTAAAGAACGTCACGAGGGGCGCTTAGAGGCACGCAGATTCAAACGTCGCAACCGCAAGGCACGTAAAGCACACAAAGCTAAGCGCGAAAGAATGCTTGCTGCGTGGCGATGGGCTGAACGTCAAGAACGGCGTAACCATGAGGTAGCTGTAGATGTACTAGgaaga                                                                                                       <  1:309353‑M2/200‑1 (MQ=255)`  
                                                                                                                                                                                                `aatccgcatcctgcaagcgcgtatctatgagcaattaacgattgacctctgggaagacgcagaagacttgctcaatgaatacttggaggaagtcgCACGACAGAAAGAAATTGACCGCGCTAAGGCCCGTAAAGAACGTCACGAGGGGCGCTTAGAGGCACGCAGATTCAAACGTCGCAACCGCAAGGCACGTAAAGCACACAAAGCTAAGCGCGAAAGAATGCTTGCTGCGTGGCGATGGGCTGAACGTCCAGAACGGCGTAACCATGAGGTAGCTGTAGATGTACTAGGAAGAACCAAt                                                                                                 >  1:595142‑M2/96‑301 (MQ=255)`  
                                                                                                                                                                                                   `ccgcatcctgcaagcgcgtatctatgagcaattaacgattgacctctgggaagacgcagaagacttgctcaatgaatacttggaggaagtcgCACGACAGAAAGAAATTGACCGCGCTAAGGCCCGTAAAGAACGTCACGAGGGGCGCTTATAGGCACGCAGATTCAAACGTCGCAACCGCAAGGCACGTAAAGCACACAAAGCTAAGCGCGAAAGAATGCTTGCTGCGTGGCGATGGGCTGAACGTCAAGAACGGCGTAACCATGAGGTAGCTGTAGATGTACTAGgaagaacctgtctc                                                                                              >  1:222289‑M2/93‑295 (MQ=255)`  
                                                                                                                                                                                                          `ctgcaagcgcgtatctatgagcaattaacgattgacctctgggaagacgcagaagacttgctcaatgaatacttggaggaagtcgCACGACAGAAAGAAATTGACCGCGCTAAGGCCCGTAAAGAACGTCACGAGGGGCGCTTAGAGGCACGCAGATTCAAACGTCGCAACCGCAAGGCACGTAAAGCACACAAAGCTAAGCGCGAAAGAATGCTTGCTGCGTGGCGATGGGCTGAACGTCAAGAAcggcgtaaccatgaggtagctgtagatgctgtctcttatacacatctccgagccc                                                                                       >  1:39469‑M2/86‑274 (MQ=255)`  
                                                                                                                                                                                                          `ctgcaagcgcgtatctatgagcaattaacgattgacctctgggaagacgcagaagacttgctcaatgaatacttggaggaagtcgCACGACAGAAAGAAATTGACCGCGCTAAGGCCCGTAAAGAACGTCACGAGGGGCGCTTAGAGGCACGCAGATTCAAACGTCGCAACCGCAAGGCACGTAAAGCACACAAAGCTAAGCGCGAAAGAATGCTTGCTGCGTGGCGATGGGCTGAACGTCAAGAACGGCGTAACCATGAGGTAGCTGTAGATGTACTAGGAAGAACCAATAACGCTATGc                                                                                       <  1:507570‑M2/216‑1 (MQ=255)`  
                                                                                                                                                                                                           `tgcaagcgcgtatctatgagcaattaacgattgacctctgggaagacgcagaatacttgctcaatgaatacttggaggaagtcgCACGACAGAAAGAAATTGACCGCGCTAAGGCCCGTAAAGAACGTCACGAGTGGCGCTTAGAGGCACGCAGATACAAACGTCGCAACCGCAAGGCACGTAAAGCACACAAAGCTAAGCGCGAAAGAATGCTTGCTGCGTGGCGATGGGCTGAACGTCAAGAACGGCGTAACCATGAGGTAGCTGTAGATGTACTAGGAAGAACCAATAACGCTATGct                                                                                      <  1:265653‑M2/217‑1 (MQ=255)`  
                                                                                                                                                                                                            `gcaagcgcgtatctatgagcaattaacgattgacctctgggaagacgcagaagacttgctcaatgaatacttggaggaagtcgCACGACAGAAAGAAATTGACCGCGCTAAGGCCCGTAAAGAACGTCACGAGGGGCGCTTAGAGGCACGCAGATTCAAACGTCGCAACCGCAAGGCACGTAAAGCACACAAAGCTAAGCGCGAAAGAATGCTTGCTGCGTGGCGATGGGCTGAACGTCAAGAACGGCGTAACCATGAGGTAGCTGTAGATGTACTAGGAAGAACCAATAACGCTATGctc                                                                                     <  1:136252‑M2/218‑1 (MQ=255)`  
                                                                                                                                                                                                                `gcgcgtatctatgagcagttaacgattgacctctgggaagacgcagaagacttgctcaatgaatacttggaggaagtcgCACGACAGAAAGAAATTGACCGCGCTAAGGCCCGTAAAGAACGTCACGAGGGGCGCTTAGAGGCACGCAGATTCAAACGTCGCAACCGCAAGGCACGTAAAGCACACAAAGCTAAGCGCGAAAGAATGCTTGCTGCGTGGCGATGGGCTGAACGTCAAGAACGGCGTAACCATGAGGTAGCTGTAGATGTACTAGGAAGAACCAATAACGCTATGCTCTggg                                                                                 <  1:400756‑M2/222‑1 (MQ=255)`  
                                                                                                                                                                                                                  `gcgtatctatgagcaattaacgattgacctctgggaagacgcagaagacttgctcaatgaatacttggaggaagtcgCACGACAGAAAGAAATTGACCGCGCTAAGGCCCGTAAAGAACGTCACGAGGGGCGCTTAGAGGCACGCAGATTCAAACGTCGCAACCGCAAGGCACGTAAAGCACACAAAGCTAAGCGCGAAAGAATGCTTGCTGCGTGGCGATGGGCTGAACGTCAAGAACGGCGTAACCATGAGGTAGCTGTAGATGTACTAGGAAGAACCAATAACGCTATGCTCTGGGTc                                                                               >  1:30194‑M2/78‑301 (MQ=255)`  
                                                                                                                                                                                                                     `tatctatgagcaattaacgattgacctctgggaagacgcagaagacttgctcaatgaatacttggaggaagtcgCACGACAGAAAGAAATTGACCGCGCTAAGGCCCGTAAAGAACGTCACGAGGGGCGCTTAGAGGCACGCAGATTCAAACGTCGCAACCGCAAGGCACGTAAAGCACACAAAGCTAAGCGCGAAAGAATGCTTGCTGCGTGGCGATGGGCTGAACGTCAAGAACGGCGTAACCATGAGGTAGCTGTAGATGTACTAGGAAGAACCAATAACGCTATGCTCTGGGTCAAc                                                                            <  1:341043‑M2/227‑1 (MQ=255)`  
                                                                                                                                                                                                                       `tctatgagcaattaacgattgacctctgggaagacgcagaagacttgctcaatgaatacttggaggaagtcgCACGACAGAAAGAACTTGACCGCGCTAAGGCCCGTAAAGAACGTCACGAGGGGCGCTTAGAGGCACGCAGATTCAAACGTCGCAACCGCAAGGCACGTAAAGCACACACAGCTAAGCGCGAAAGCATGCTTGCTGCGTGGCGCTGGGCTGAACGTCAAGAACGGCGTAACCATGAGGTCGCTGTAGCTGTACTAGGAAGAACCACtaacgctatgctctctgtctctta                                                                          >  1:743951‑M2/73‑291 (MQ=255)`  
                                                                                                                                                                                                                       `tctatgagcaattaacgattgacctctgggaagacgcagaagacttgctcaatgaatacttggaggaagtcgCACGACAGAAAGAAATTGACCGCGCTAAGGCCCGTAAAGAACGTCACGAGGGGCGCTTAGAGGCACGCAGATTCAAACGTCGCAACCGCAAGGCACGTAAAGCACACAAAGCTAAGCGCGAAAGAATGCTTGCTGCGTGGCGATGGGCTGAACGTCAAGAACGGCGTAACCATGAGGTAGCTGTAGATGTACTAGGAAGAACCAATAACGTTATGCTCTGGGTCAAcaa                                                                          >  1:31748‑M2/73‑300 (MQ=255)`  
                                                                                                                                                                                                                        `ctatgagcaattaacgattgacctctgggaagacgcagaagacttgctcaatgaatacttggaggaagtcgAACGACAGAAAGAAATTGACCGCGCTAAGGCCCGTAAAGAACGTCACGAGGGGCGCTTAGAGGCACGCAGATTCAAACGTCGCAACCGCAAGGCACGTAAAGCACACAAAGCTAAGCGCGAAAGAATGCTTGCTGCGTGGCGATGGGCTGAACGTCAAGAACGGCGTAACCATGAGGTAGCTGTAGATGTACTAGGAAGAACCAATAACGCTATGCTCTGGGTCAACATg                                                                         <  1:603023‑M2/230‑1 (MQ=255)`  
                                                                                                                                                                                                                                 `attaacgattgacctctgggaagacgcagaagacttgctcaatgaatacttggaggaagtcgCACGACAGAAAGAAATTGACCGCGCTAAGGCCCGTAAAGAACGTCACGAGGGGCGCTTAGAGGCACGCAGATTCAAACGTCGCAACCGCAAGGCACGTAAAGCACACAAAGCTAAGCGCGAAAGAATGCTTGCTGCGTGGCGATGGGCTGAACGTCAAGAACGGCGTAACCATGAGGTAGCTGTAGATGTACTAGGAAGAACCAATAACGCTATGCTCTGGGTCAACATGTTCTCTggg                                                                >  1:200815‑M2/63‑301 (MQ=255)`  
                                                                                                                                                                                                                                  `ctcggagatgtgtataagagacaggcagaagacttgctcaatgaatacttggaggaagtcgCACGACAGAAAGAAATTGACCGCGCTAAGGCCCGTAAAGAACGTCACGAGGGGCGCTTAGAGGCACGCAGATTCAAACGTCGCAACCGCAAGGCACGTAAAGCACACAAAGCTAAGCGCGAAAGAATGCTTGCTGCGTGGCGATGGGCTGAACGTCAAGAACGGCGTAACCATGAGGTAGCTGTAGATGTACTAGGAAGAACCAATAACGCTATGCTCTGGGTCAACATGTTCTCTgggg                                                               <  1:492466‑M2/240‑1 (MQ=255)`  
                                                                                                                                                                                                                                           `gacctctgggaagacgcagaagacttgctcaatgaatacttggaggaagtcgCACGACAGAAAGAAATTGACCGCGCTAAGGCCCGTAAAGAACGTCACGAGGGGCGCTTAGAGGCACGCAGATTCAAACGTCGCAACCGCAAGGCACGTAAAGCACACAAAGCTAAGCGCGAAAGAATGCTTGCTGCGTGGCGATGGGCTGAACGTCAAGAACGGCGTAACCATGAGGTAGCTGTAGATGTACTAGGAAGAACCAATAACGCTATGCTCTGGGTCAACATGTTCTCTGGggactttccgt                                                      >  1:408223‑M2/53‑297 (MQ=255)`  
                                                                                                                                                                                                                                             `cctctgggcagacgcagaagacttgctcaatgaatacttggaggaagtcgCACGACAGAAAGAAATTGACCGCGCTAAGGCCCGTAAAGAACGTCACGAGGGGCGCTTAGAGGCACGCAGATTCAAACGTCGCAACCGCAAGGCACGTAAAGCACACAAAGCTAAGCGCGAAAGAATTGTTGCTGCGTGGCGATGGGCTTAACGTCCAAGACGGCGTAAACATGAGGGAGCTGTAGATGTACGAAGAAGAACAAATAACGCTATGCTCTGGGTCAAGATCTTCCCTGGGGactttacgtcg                                                    >  1:41656‑M2/51‑296 (MQ=255)`  
                                                                                                                                                                                                                                                `ctgggaagacgcagaagacttgctcaatgaatacttggaggaagtcgCACGACAGAAAGAAATTGACCGCGCTAAGGCCCGTAAAGAACGTCACGAGGGGCGCTTAGAGGCACGCAGATTCAAACGTCGCAACCGCAAGGCACGTAAAGCACACAAAGCTAAGCGCGAAAGAATGCTTGCTGCGTGGCGATGGGCTGAACGTCAAGAACGGCGTAACCATGAGGTAGCTGTAGATGTACTAGGAAGAACCAATAACGCTATGCTCTGGGGCAACATGTTCTATGGGGACTTTAAGGCGCtt                                                 >  1:820856‑M2/48‑301 (MQ=255)`  
                                                                                                                                                                                                                                                    `gaagacgccgaagacttgctcaatgaatacttggaggaagtcgCACGACAGAAAGAAATTGACCGCGCTAAGGCCCGTAAATAACGTCACGCGGGGCGCTTAGAGGCACGCAGATTCCAACGTCGCAACCGCAAGGCACGTAAAGCACACAAAGCTAAGCGCGCAAGAATTCTTGCTGCTTGGCGATGGGCTTAACGTCCAGAACGGCGTAACCATGAGGTAGCTGTAGATGTTCTAGGAAGAACCATTCACTCTTTGCTCTGGGTCAACCTGTTCTCTGGGGACTTTAATGCGCTTGTgg                                             >  1:632446‑M2/44‑301 (MQ=255)`  
                                                                                                                                                                                                                                                           `cagaagacttgctcaatgaatacttggaggaagtcgCACGACAGAAAGAAATTGACCGCGCTAAGGCCCGTAAAGAATGTCACGAGGGGCGCTTAGAGGCACGCAGATTCAAACGTCGCAACCGCAAGGCACGTAAAGCACACAAAGCTAAGCGCGAAAGAATGCTTGCTGCGTGGCGATGGGCTGAACGTCAAGAACGGCGTAACCATGAGGTAGCTGTAGATGTACTAGGAAGAACCAATAACGCTATGCTCTGGGTCAACATGTTCTCTGGGGACTTTAAGGCGCTTGAGGAACGAAt                                      <  1:707241‑M2/265‑1 (MQ=255)`  
                                                                                                                                                                                                                                                            `gggaaaagggaccagtgaaaaattggaggaaggtgGAAGAAAAAAAGGAATTGAACGCGCTAAGGCCCGGAAAGAACGTCAAGAAGGGCGCTTAGAGGCACGCAGATTCAAACGTCGCAACCGCAAGGCACGTAAAGCACACAAAGCTAAGCGCGAAAGAATGCTTGCTGCGTGGCGATGGGCTGAACGTCAAGAACGGCGTAACCATGAGGTAGCTGTAGATGTACTAGGAAGAACCAATAACGCTATGCTCTGGGTCAACATGTTCTCTGGGGACTTTAAGGCGCTTGAGGAACGAATc                                     <  1:371604‑M2/266‑1 (MQ=255)`  
                                                                                                                                                                                                                                                             `gaagacttgctcaatgaatacttggaggaagtcgCACGACAGAAAGAAATTGACCGCGCTAAGGCCCGTAAAGAACGTCACGAGGGGCGCTTAGAGGCACGCAGATTCAAACGTCGCAACCGCAAGGCACGTAAAGCACACAAAGCTAAGCGCGAAAGAATGCTTGCTGCGTGGCGATGGGCTGAACGTCAAGAACGGCGTAACCATGAGGTAGCTGTAGATGTACTAGGAAGAACCAATAACGCTATGCTCTGGGTCAACATGTTCTCTGGGGACTTTAAGGCGCTTgaggaactgtctc                                    >  1:641335‑M2/35‑295 (MQ=255)`  
                                                                                                                                                                                                                                                                     `gacagatgaatacttggaggaagtcgCACGACAGAAAGAAATTGACCGCGCTAAGGACCGTAAAGAACGTCACGAGGGGCGCTTAGAGGCACGCAGATTCAAACGTCGCAACCGCAAGGCACGTAAAGCACACAAAGCTAAGCGCGAAAGAATGCTTGCTGCGTGGCGATGGGCTGAACGTCAAGAACGGCGTAACCATGAGGTAGCTGTAGATGTACTAGGAAGAACCAATAACGCTATGCTCTGGGTCAACATGTTCTCTGGGGACTTTAAGGCGCTTGAGGAACGAATCGCGCTGCAc                            <  1:579820‑M2/275‑1 (MQ=255)`  
                                                                                                                                                                                                                                                                        `aaatgaatacttggaggaagtcgCACGACAGAAAGAAATTGACCGCGCTAAGGCCCGTAAAGAACGTCACGAGGGGCGCTTAGAGGCACGCAGATTCAAACGTCGCAACCGCAAGGCACGTAAAGCACACAAAGATAAGCGCGAAAGAATGCTTGCTGCGTGGCGATGGGCTGAACGTCAAGAACGGCGTAACCATGAGGTAGCTGTAGATGTACTAGGAAGAACCAATAACGCTATGCTCTGGGTCAACATGTTCTCTGGGGACTTTAAGGCGCTTGAGGAACGAATCGCGCTGCACTgg                         <  1:672855‑M2/278‑1 (MQ=255)`  
                                                                                                                                                                                                                                                                             `aatacttggaggaagtcgCACGACAGAAAGAAATTGACCGCGCTAAGGCCCGTAAAGAACGTCACGAGGGGCGCTTAGAGGCACGCAGATTCAAACGTCGCAACCGCAAGGCACGTAAAGCACACAAAGCTAAGCGCGAAAGAATGCTTGCTGCGTGGCGATGGGCTGAACGTCAAGAACGGCGTAACCATGAGGTAGCTGTAGATGTACTAGGAAGAACCAATAACGCTATGCTCTGGGTCAACATGTTCTCTGGGGACTTTAAGGCGCTTGAGGAACGAATCGCGCTGCACTGGCGTaa                    <  1:271413‑M2/283‑1 (MQ=255)`  
                                                                                                                                                                                                                                                                               `tacttggaggaagtcgCACGACAGAAAGAAATTGACCTCGCTAAGGCCCGTAAAGAACGTCACGAGGGGCGCTTAGAGGCACGCAGATTCAAACGTCGCAACCGCAAAGCACGTAAAGCACACAAAGCTAAGCGCGAAAGAATGCTTGCTGCGTGGCGATGGGCTGAACGTCAAGAACGGCGTAACCATGAGGTAGCTGTAGATGTACTCGGAAGAACCAATAACGCTATGCTCTGGGCCAACATGTTCTCTGGGGACTTTAAGGCGCTTGAGGAACGCATCGCGCTGCACTGGCGTCATg                  >  1:229853‑M2/17‑301 (MQ=255)`  
                                                                                                                                                                                                                                                                                `acttggaggaagtcgCACGACAGAAAGAAATTGACCGCGCTAAGGCCCGTAAAGAACGTCACGAGGGGCGCTTAGAGGCACGCAGATTCAAACGTCGCAACCGCAAGGCACGTAAAGCACACAAAGCTAAGCGCGAAAGAATGCTTGCTGCGTGGCGATGGGCTGAACGTCAAGAACGGCGTAACCATGAGGTAGCTGTAGATGTACTAGGAAGAACCAATAACGCTATGCTCTGGGTCAACATGTTCTCTGGGGACTTTAAGGCGCTTGAGGAACGAATCGCGCTGCACTGGCGTAATGc                 <  1:192539‑M2/286‑1 (MQ=255)`  
                                                                                                                                                                                                                                                                                `acttggaggaagtcgCACGACAGAAAGAAATTGACCGCGCTAAGGCCCGTAAAGAACGTCACGAGGGGCGCTTAGAGGCACGCAGATTCAAACGTCGCAACCGCAAGGCACGTAAAGCACACAAAGATAAGCGCGAAAGAATGCTTGCTGCGTGGCGATGGGCTGAACGTCAAGAACGGCGTAACCATGAGGTAGCTGTAGATGTACTAGGCAAAACCAATACCGCGATGCTCTGGTTCAACATGTTCTCTGGGGACTTTAAGGCGATTGAGGCACGAATCGCGCTGCACTGGCGTAAtgg                 >  1:522547‑M2/16‑300 (MQ=255)`  
                                                                                                                                                                                                                                                                                `acttggaggaagtaaCACAACAGAAAGAAATTGAACGAGCTAAGGCCCGTAAAGAACGTCACGAAGGGCGCTTAGAGGCACGCAGATTCAAACGTCGCAACCGAAAGGCACGTAAAGCACGCAAAGCTAAGCGCGAAAGAATGCTTGCTGCGTGGCGATGGGCTGGACGTCAAGAACGGCGTAACCATGAGGTAGCTGTAGATGTACTATGAAGTACCAATAACGCTATGCTCTGGGTCAACATGTTCTCTGGGGACTTTAAGGCGCTTGAGGAACGAATCGCGCTGCACTGGCGTAATGc                 <  1:478952‑M2/286‑1 (MQ=255)`  
                                                                                                                                                                                                                                                                                 `cttggaggaagtcgCACGACAGAAAGAAATCGACCGCGCTAAGCCCCGTAAAGAACGTCACGAGGGGCGCTTAGAGGCACGCAGATTCAAACGTCGCAACCGCAAGGCACGTAAAGCACACAAAGCTAAGCGCGAAAAAATGCTTGCTGCGTGGCGATGGGCCGAACGTCAAGAACGGCGTAACCATGAGGTAGCTGTAGATGTACTAGGAAGAACCAATAACGCTATGCTCTGGGTCAACATGTTCTCTGGGGACTTTCAGGCGCCTGAGGTACGAATAGCGCCGCACTGGCGTAATGCt                >  1:332984‑M2/15‑301 (MQ=255)`  
                                                                                                                                                                                                                                                                                    `ggaggaagtcgCACGACAGAAAGAAATTGACCGCGCTAAGGCCCGTAAAGAACGTCACGAGGGGCGCTTAGAGGCACGCAGATTCAAACGTCGCAACCGCCAGGCACGTAAAGCACACAAAGCTAAGCGCGAAAGAATGCTTGCTGCGTGGCGATGGGCTGAACGTCAAGAACGGCGTAACCATGCGGTATCTGTAGATGTACTAGGAAGAACCAATAACGCTATGCTCTGGGTCAACATGTTCTCTGGGTCCTTTACGGCGCTTGAGGCACTAATCGCTCTTCACTGTCGTACTGCTGAc             >  1:751045‑M2/12‑301 (MQ=255)`  
                                                                                                                                                                                                                                                                                            `tcgCACGACAGACAGAAATTGACCGCGCTAAGGCCCGTAAAGAACGTCACGAGGGGCGCTTAGAGGCACGCAGATTCAAACGTCGCAACCGCAAGGCACGTAAAGCACACAAAGCTAAGCGCGAAAGAATGCTTGCTGCGTGGCGATGGGCTGAACGTCAAGAACGGCGTAACCATGAGGTAGCTGTAGATGTACTAGGAAGAACCAATAACGCTATGCTCTGGGTCAACATGTTCTCTTGGGCCTTTAAGGCGCTTGCGGAACGAATCGCGCTGCACTTGCGTAATGCCGACCGGATGGc     >  1:785330‑M2/4‑301 (MQ=255)`  

                                                                                                                                                                                                                                                                                               `|`                                                                                                                                                                                                                                                                                                              
`CAATGATGCCCATTATAAATACGCTGAGATTGCAAGCGACATCATTGATTGCAATTCGGATGAGCATGATGAGTTAACTGGATGGGATGGTGAGTTTGTTGAAACTTGTAAACTAATCCGCAAGTTCTTTGAGGGCATCGCCTCATTCGACATGCATAGCGGGAACATCATGTTCTCAAATGGAGACGTACCATACATCACCGACCCGGTATCATTCTCGCAGAAGAAAGACGGTGGCGCATTCAGCATCGACCCTGAGGAACTCATCAAGGAAGTCGAGGAAGTCGCACGACAGAAAGAAATTGACCGCGCTAAGGCCCGTAAAGAACGTCACGAGGGGCGCTTAGAGGCACGCAGATTCAAACGTCGCAACCGCAAGGCACGTAAAGCACACAAAGCTAAGCGCGAAAGAATGCTTGCTGCGTGGCGATGGGCTGAACGTCAAGAACGGCGTAACCATGAGGTAGCTGTAGATGTACTAGGAAGAACCAATAACGCTATGCTCTGGGTCAACATGTTCTCTGGGGACTTTAAGGCGCTTGAGGAACGAATCGCGCTGCACTGGCGTAATGCTGACCGGATGGCTAT  >  NC_001604.1/2455‑3042`

  

Alignment Legend

Aligned base mismatch/match (shaded by quality score): `ATCG/ATCG` `< 3 ≤` `ATCG/ATCG` `< 10 ≤` `ATCG/ATCG` `< 24 ≤` `ATCG/ATCG` `< 36 ≤` `ATCG/ATCG` `< 38 ≤` `ATCG/ATCG`

Unaligned base: `atcg`    Masked matching base: `atcg`    Alignment gap: `‑`    Deleted base: `‑`
